# Reversible optical control of F_1_F_o_‐ATP synthase using photoswitchable inhibitors

**DOI:** 10.1002/1873-3468.12958

**Published:** 2018-02-01

**Authors:** Bianca Eisel, Felix W.W. Hartrampf, Thomas Meier, Dirk Trauner

**Affiliations:** ^1^ Department of Structural Biology Max Planck Institute of Biophysics Frankfurt am Main Germany; ^2^ Department of Life Sciences Imperial College London UK; ^3^ Department of Chemistry University of Munich Germany; ^4^ Department of Chemistry New York University NY USA

**Keywords:** photopharmacology, *Yarrowia lipolytica* F_1_F_o_‐ATP synthase

## Abstract

F_1_F_o_‐ATP synthase is one of the best studied macromolecular machines in nature. It can be inhibited by a range of small molecules, which include the polyphenols, resveratrol and piceatannol. Here, we introduce Photoswitchable Inhibitors of ATP Synthase, termed PIAS, which were synthetically derived from these polyphenols. They can be used to reversibly control the enzymatic activity of purified yeast *Yarrowia lipolytica* ATP synthase by light. Our experiments indicate that the PIAS bind to the same site in the ATP synthase F_1_ complex as the polyphenols in their *trans* form, but they do not bind in their *cis* form. The PIAS could be useful tools for the optical precision control of ATP synthase in a variety of biochemical and biotechnological applications.

F_1_F_o_‐type ATP synthase is a membrane‐embedded, macromolecular rotary machine that discharges the transmembrane electrochemical ion gradient to synthesize ATP from ADP and inorganic phosphate (P_i_). This key metabolic enzyme uses a unique mechanochemical rotary mechanism to produce the bulk amount of universal energy currency ATP in all living cells, but it is also able to operate in reverse, hydrolyzing ATP, to establish ion gradients by exploiting the energy released from hydrolysis of ATP 1.

In eukaryotes, the ATP synthase is embedded in the inner membrane of mitochondria or in the thylakoid membranes of chloroplasts, while in bacteria and archaea, it is located in the cytoplasmic membrane. In all organisms, the ATP synthase shares an overall highly conserved architecture consisting of a water soluble F_1_ complex (subunits α_3_β_3_γδε) and a membrane‐intrinsic F_o_ complex (ab_2_c_8–17_)^2^, ^3^, ^4^ joined together by a central stalk (subunits γ and ε) and a peripheral stalk (subunits b_2_ and δ). The α_3_β_3_ subunits envelop the central stalk γ subunit which by itself introduces an inherent asymmetry into the F_1_ headpiece. The lower part of the γ and ε subunit is in contact with the membrane‐embedded F_o_ rotor, formed by a number of identical copies of c‐subunit, called the c‐ring. Recent advances in structural biology have provided new insights into the structure and dynamics of completely assembled complexes of ATP synthase. In particular, it includes also valuable structural information about the previously less well‐characterized F_o_ stator complex in the membrane, its outer stalk region as well as the structural basis of dimerization of mitochondrial ATP synthases 5, 6, 7, 8. For example, the yeast *Yarrowia lipolytica* F_1_F_o_‐ATP synthase dimer consists of a total of more than 60 different proteins, which in mitochondria form a dimeric ATP synthase of about 1.25 MDa in size and play an important role in the determination of cristae morphology of the inner mitochondrial membrane 8.

From an enzymatic functional point of view, the F_1_ complex is the catalytic, ATP‐producing or ‐consuming mechanochemical motor, while the F_o_ complex represents the electrical motor that generates torque by dissipating the ion gradient by ion translocation. ATP synthesis is driven by the flow of ions through F_o_, leading to a rotation of the c‐(rotor) ring, which transmits rotation into F_1_ via the γ subunit. It is the intrinsically asymmetric γ subunit that finally elicits sequential conformational changes in the three catalytic β subunits, leading to ATP synthesis 9, 10.

Inhibitors of ATP synthase have played an important role in the discovery and biochemical characterization of ATP synthases over many decades (for a review, see 11). The ATP synthesis or hydrolysis can be inhibited by a range of compounds that bind, for example, to the rotor–stator interface region within the F_1_ headpiece thereby interfering either with the rotational ATP‐ synthesizing or ATP‐hydrolyzing mechanism, or both 11, 12. Among them is one particular class of natural products, known as polyphenols, which includes stilbene derivatives, such as resveratrol and piceatannol, and flavonoids, such as quercetin (Fig. 1A). Natural polyphenols are found in grapes, peanuts, berries, and red wine. Due to their pharmacokinetic properties and relatively low affinities to human ATP synthases, they are nontoxic at concentrations found in their natural sources. They have been shown to extend the life span of simple organisms 13, but their value in human medicine remains to be determined.

**Figure 1:**
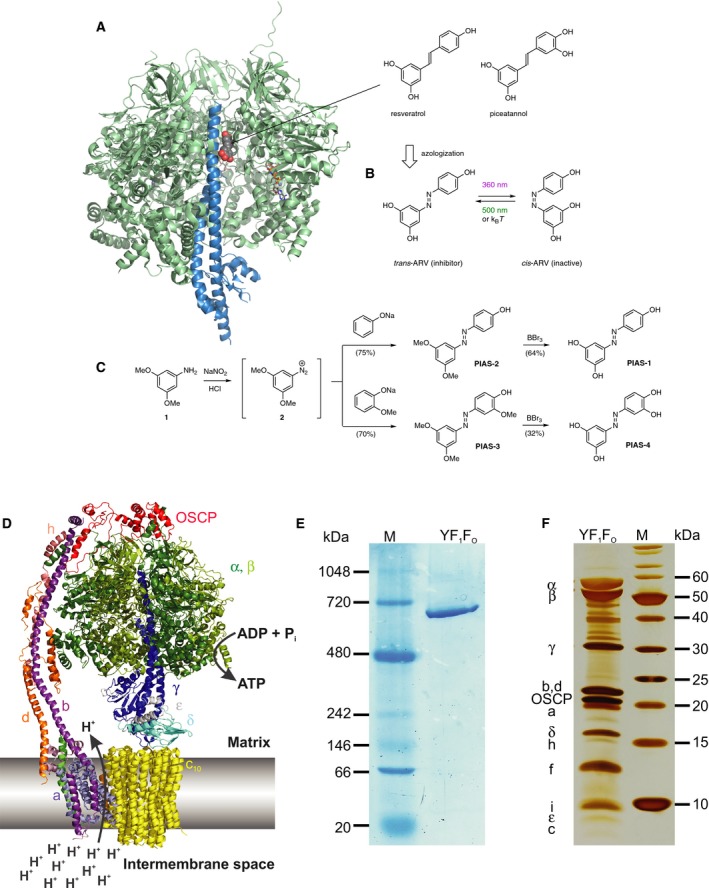
Design and synthesis of the PIAS. (A) Structure of resveratrol bound to bovine F_1_ ATP synthase (from PDB 2JIZ) shown in cartoon representation. Green: α and β subunits of F_1_. Blue: γ subunit. The β_DP_‐site containing subunit is removed to provide an unobstructed view of resveratrol wedged between the γ rotor subunit and the α, β stator subunits. Resveratrol (CPK colors, sphere model) is bound in two overlapping orientations. ATP is shown as a stick model in the β_TP_ site. (B) Azologization of resveratrol affords PIAS‐1. (C) Chemical synthesis of PIAS‐1–4. (D) Cartoon representation of the *Yarrowia lipolytica* F_1_F_o_ ATPase (8), and (D) Its biochemical characterization by (E) clear native PAGE, Coomassie‐stained gel and (F) SDS PAGE, silver‐stained gel.

Stilbenes closely resemble the azobenzenes, a very well‐established class of synthetic photoswitches 14. This suggested that the replacement of the stilbene moiety with an isosteric azobenzene (“azologization”) could convert resveratrol and piceatannol into photoswitchable inhibitors whose activity could be turned ON and OFF by light (15, Fig. 1B). Azobenzenes undergo fast photoisomerization from the thermodynamically more stable, linear *trans* to the more unstable, bent *cis* form upon irradiation with UV‐A or visible light 16. The *cis* form reverts thermally or can be switched back actively by a different wavelength of light. The wavelength needed for photoisomerization and the rate of thermal reversion can be tuned by modification of the azobenzene chromophore 17, 18. Unlike caged compounds, whose uncaging is irreversible, azobenzenes can be switched ON and OFF repeatedly and over thousands of cycles 16. They have played a central role in the development of photopharmacology, which is an attempt to control biological function with artificial photoswitches 19, 20, 21. This concept has been applied to biological targets as diverse as ion channels, G‐protein coupled receptors, enzymes, and microtubules 22, 23, 24, 25, 26, 27, 28.

We now report on the extension of photopharmacology to F‐type ATP synthases. To this end, we introduce Photoswitchable Inhibitors of ATP Synthase, termed PIAS 1–4 (Fig. 1). We demonstrate that ATP hydrolysis of our test system, the purified yeast *Y. lipolytica* F_1_F_o_‐ATP synthase (Fig. 1D–F), can be optically switched ON and OFF *in vitro* using these molecules. Our results provide a blueprint for the development of precision tools to spatiotemporally control ATP levels and pH gradients in biological systems.

## Materials and methods

### Chemical synthesis

All reactions of the chemical synthesis of PIAS 1–4 were performed with standard Schlenk techniques under an atmosphere of nitrogen in ovendried glassware (100 °C oven temperature) that was further dried using a heat gun (set to 650 °C) for all water‐sensitive reactions. Dichloromethane (CH_2_Cl_2_) was distilled from calcium hydride. Reagents were purchased from Sigma‐Aldrich (Schnelldorf, Germany), TCI (Eschborn, Germany) or Acros Organics (VWR International GmbH, Darmstadt, Germany) and used without further purification. Reaction progress was monitored by analytical TLC, which was carried out using precoated glass plates (silica gel 60°F_254_) from Merck. Visualization was achieved by exposure to ultraviolet light (UV, 254 nm) where applicable followed by staining with potassium permanganate solution. Flash column chromatography was performed using Merck silica gel (40–63 μm particle size). Proton NMR (^1^H NMR) spectra were recorded on a Varian 300, Varian 400, Inova 400, or Varian 600 spectrometer. Chemical shifts (δ scale) are expressed in parts per million (p.p.m.) and are calibrated using residual protic solvent as an internal reference (CHCl_3_: δ = 7.26 p.p.m., CD_3_OD: δ = 3.31 p.p.m.). Data for ^1^H NMR spectra are reported as follows: chemical shift (p.p.m.; multiplicity, coupling constants (Hz), integration). Couplings are expressed as: s = singlet, d = doublet, t = triplet, m = multiplet, or combinations thereof. Carbon NMR (^13^C NMR) spectra were recorded on the same spectrometers at 75, 100, and 150 MHz (± 1 MHz variance). Carbon chemical shifts (δ scale) are also expressed in p.p.m. and are referenced to the central carbon resonances of the solvents (CDCl_3_: δ = 77.16 p.p.m., CD_3_OD: δ = 49.00 p.p.m.). IR spectra were recorded on a Perkin Elmer Spectrum BX II (FTIR System) equipped with an attenuated total reflection (ATR) measuring unit. IR data are reported in frequency of absorption (cm^−1^). Mass spectroscopy (MS) experiments were performed on a Thermo Finnigan MAT 95 (electron ionization, EI) or on a Thermo Finnigan LTQ FT (electrospray ionization, ESI) instrument.

### 4‐((3,5‐dimethoxyphenyl)diazenyl)phenol (PIAS‐2)

To an ice‐cold solution of 3,5‐dimethoxyaniline (306 mg, 2.00 mmol, 1.00 eq.) in THF (5 mL) and HCl (1 m, 6 mL), an aqueous solution (5 mL) of sodium nitrite (166 mg, 2.40 mmol, 1.20 eq.) was added dropwise, resulting in the formation of a dark‐red suspension. After 30 min, a solution of phenol (226 mg, 2.40 mmol, 1.20 eq) in aqueous NaOH (1 m, 6 mL) was added dropwise. The mixture was stirred at 0 °C for 1 h before the bulk of the solvent was removed *in vacuo*. The residue was redissolved in ethyl acetate (15 mL) and water (15 mL). After phase separation, the aqueous phase was further extracted with ethyl acetate (3 × 20 mL). The combined organic layers were washed with water (20 mL) and aqueous saturated sodium chloride (20 mL), then dried over MgSO_4_ and concentrated under reduced pressure. Purification by flash column chromatography (5 : 1 hexane: ethyl acetate) afforded PIAS‐2 (387 mg, 1.50 mmol, 75%) as a yellow solid.

^1^H NMR (400 MHz, CD_3_OD) δ = 7.81–7.71 (m, 2H), 6.95–6.86 (m, 2H), 6.82 (d, *J *= 2.2, 2H), 6.38 (t, *J *= 2.2, 1H).

HRMS (ESI) *m*/*z* calculated for C_14_H_15_N_2_O_3_ 259.1077; found 259.1080. (M + H^+^).

Analytical data were in good agreement with literature values 29.

### 4‐((3,5‐dimethoxyphenyl)diazenyl)‐2‐methoxyphenol (PIAS‐3)

To an ice‐cold solution of 3,5‐dimethoxyaniline (306 mg, 2.00 mmol, 1.00 eq.) in THF (5 mL) and HCl (1 m, 6 mL), an aqueous solution (5 mL) of sodium nitrite (166 mg, 2.40 mmol, 1.20 eq.) was added dropwise, resulting in the formation of a dark‐red suspension. After 30 min, a solution of guaiacol (298 mg, 2.40 mmol, 1.20 eq) in aqueous NaOH (1 m, 6 mL) was added dropwise. The mixture was stirred at 0 °C for 1 h before the bulk of the solvent was removed *in vacuo*. The residue was redissolved in ethyl acetate (15 mL) and water (15 mL). After phase separation, the aqueous phase was further extracted with ethyl acetate (3 × 20 mL). The combined organic layers were washed with water (20 mL) and aqueous saturated sodium chloride (20 mL), then dried over MgSO_4_ and concentrated under reduced pressure. Purification by flash column chromatography (5 : 1 hexane : ethyl acetate) afforded PIAS‐3 (243 mg, 1.40 mmol, 70%) as a yellow solid.

^1^H NMR (400 MHz, CDCl_3_ δ = 7.61 (dt, *J *= 8.4, 1.5, 1H), 7.50 (t, *J *= 1.5, 1H), 7.10–7.08 (m, 2H), 7.06 (dd, *J *= 8.4, 1.1, 1H), 6.57 (q, *J *= 2.0, 1H), 5.95 (s, 1H), 4.00 (d, *J *= 1.1, 3H), 3.88 (d, *J *= 1.1, 6H).

HRMS (ESI) *m*/*z* calculated for C_15_H_17_O_4_N_2_ 289.1183; found 289.1187 (M + H^+^).

Analytical data were in good agreement with literature values 29.

### 5‐((4‐hydroxyphenyl)diazenyl)benzene‐1,3‐diol (PIAS‐1)

To a solution of dimethyl PIAS‐2 (77 mg, 0.30 mmol, 1.00 eq) in CH_2_Cl_2_ (8 mL), boron tribromide solution was added in CH_2_Cl_2_ (1.0 m, 2.1 mL, 2.1 mmol, 7.0 eq) dropwise at 0 °C. After warming to room temperature over 14 h, liquid chromatography‐mass spectrometry (LC‐MS) analysis indicated full conversion. Saturated aq. NaHCO_3_ (15 mL) was added at room temperature and the mixture was poured on water (10 mL). After extraction of the aq. phase with EtOAc (4 × 20 mL), the combined organic phases were washed with aqueous saturated sodium chloride (20 mL), dried and evaporated to give a black oil that was purified by column chromatography (9 : 1 CH_2_Cl_2_ : MeOH) to give PIAS‐1 as a dark‐red solid (44 mg, 0.19 mmol, 64%).

^1^H NMR (400 MHz, CD_3_OD) δ = 7.81–7.71 (m, 2H), 6.95–6.86 (m, 2H), 6.82 (d, *J *= 2.2, 2H), 6.38 (t, *J *= 2.2, 1H).

HRMS (ESI) *m*/*z* calculated for C_12_H_10_O_3_N_2_ 231.0764; found 231.0763 (M + H^+^).

Analytical data were in good agreement with literature values 29.

### 4‐((3,5‐dihydroxyphenyl)diazenyl)benzene‐1,2‐diol (PIAS‐4)

To a solution of PIAS‐3 (115 mg, 0.40 mmol, 1.00 eq) in CH_2_Cl_2_ (8 mL), boron tribromide solution was added in CH_2_Cl_2_ (1.0 m, 3.6 mL, 3.6 mmol, 9.0 eq) dropwise at 0 °C. After warming to room temperature over 14 h, LC‐MS analysis indicated full conversion. Saturated aq. NaHCO_3_ (15 mL) was added at room temperature and the mixture was poured on water (10 mL). After extraction of the aq. phase with EtOAc (4 × 20 mL), the combined organic phases were washed with aqueous saturated sodium chloride (20 mL), dried and evaporated to give a black oil that was purified by column chromatography (9 : 1 CH_2_Cl_2_ : MeOH) to give PIAS‐4 as a dark‐red amorphous solid (32 mg, 0.13 mmol, 32%).

R_f_ 0.52 (water : MeOH 3 : 2).

^1^H NMR (400 MHz, CD_3_OD) δ = 7.37–7.33 (m, 2H), 6.92–6.88 (m, 1H), 6.79 (d, *J *= 2.2, 2H), 6.37 (t, *J *= 2.2, 1H).

^13^C NMR (101 MHz, CD_3_OD) δ = 160.0, 156.1, 150.4, 147.7, 147.0, 120.2, 115.9, 107.6, 105.4, 102.1.

IR (ATR) 3258, 1675, 1601, 1379, 1284, 1156, 1001 cm^−1^.

HRMS (ESI) *m*/*z* calculated for C_12_H_11_O_4_N_2_ 247.0641; found 247.0713.

See Figs S3 and S4 for NMR spectra of PIAS‐4.

### Purification of *Yarrowia lipolytica* F_1_F_o_‐ATP synthase

Monomeric form of *Y. lipolytica* F_1_F_o_‐ATP synthase was purified as described in 8. Briefly, the ATP synthase was isolated from mitochondria prepared from large‐scale *Y. lipolytica* cultures 30. The isolation, solubilization, and collection of solubilized material from mitochondrial membranes were carried out as previously described 31. After the removal of complex I by metal affinity purification 31, glycerol was added to a final concentration of 20% (v/v) to the solubilized membranes, which were rapidly frozen in liquid nitrogen for storage at −80 °C. The solubilizate was thawed on ice and supplied with 50 mm MgCl_2_. To the slow stirring suspension on ice, 3% (w/w) polyethylene glycol (PEG) 6000 was added from a 50% (w/w) stock solution to induce protein precipitation. After 15 min of continued stirring on ice, the precipitated proteins were removed by centrifugation (15 min and 20 000 ***g*** at 4 °C). The ATP synthase‐containing fraction was then precipitated by the increase in the polyethylene glycol 6000 concentration to 6% (w/w), again with continuous stirring on ice for 15 min and then collected by centrifugation for 15 min, 20 000 ***g*** at 4 °C. The pellet was then dissolved in 2 mL of buffer A [30 mm 3‐(*N*‐morpholino)‐propanesulfonic acid (MOPS/NaOH pH 7.4), 4 mm MgCl_2_, 2 mm EDTA and 0.1% (w/v) DDM], the sample was applied on a density‐based discontinuous glycerol gradient (1 mL steps with 15, 20, 25, 28, 30, 35, 40, 45, 50% glycerol in buffer A) and run for 16 h at 4 °C and at 151 177 ***g*** in a SW40 rotor (Beckman Coulter, Indianapolis, IN, USA). After the run, 1 mL fractions were collected from top using a pipette and the ATP synthase‐containing fractions, as judged by high‐resolution Clear Native PAGE (hrCN‐PAGE 32), were pooled and directly loaded onto an anion exchange chromatography using a POROS GoPure HQ 50 anion exchange column (Life Technologies, ThermoFisher Scientific, Waltham, MA, USA), which was previously equilibrated with one column volume (CV) of buffer A, using an ÄKTAexplorer chromatography system (GE Healthcare, Munich, Germany). The column was then washed with 1 CV of buffer A and the *Y. lipolytica* ATP synthase was eluted by a continuous gradient using buffer B (buffer A with 1 m NaCl). The ATP synthase as judged by high‐resolution Clear Native PAGE (hrCN‐PAGE 32) was concentrated to 1 mg·mL^−1^ by ultrafiltration using Vivaspin PES membranes with a molecular weight cutoff of 100 kDa at 1500 ***g*** and 4 °C to 2 mL final volume. The protein concentration was determined using the bicinchoninic acid (BCA) method (Pierce; ThermoFisher Scientific). Bovine serum albumin was used as a standard between 2 and 2000 μg·mL^−1^.

### Determination of ATP hydrolysis activity using the malachite green assay

The ATP hydrolysis activity was determined using the malachite green assay as described in 33, 34, 35. Briefly, 1 mg·mL^−1^ *Y. lipolytica* ATP synthase protein solution was prepared in a reaction tube and supplemented with 10 μg·mL^−1^ cardiolipin and 40 μg·mL^−1^ yeast extract lipids and diluted in the reaction buffer (50 mm Tricine‐NaOH pH 8.0, 5 mm MgCl_2_; 150 μL per experiment). The malachite green stock reagent (320 μL per experiment) was prepared [0.08% (w/v) malachite green: 2.3% (v/v) polyvinyl alcohol: 5.7% (w/v) ammonium molybdate: 6 N HCl in distilled water (ratio 2 : 1 : 1 : 2)] and incubated for 30 min at room temperature before use. A time course for ATPase activity was measured, taking sample aliquots from the reaction tube and stopping the reaction after four time points, in 1‐min intervals: Each reaction was started by the addition of 5 mm Na_2_‐ATP (pH 7.4) using a 0.2 m stock solution. At each taken time point, an aliquot of 20 μL of reaction mix was transferred into 80 μL malachite green reagent and immediately mixed. After 75 seconds, each reaction was quenched by the addition of 34% (w/v) sodium citrate. Each time course was measured in triplicates; a calibration curve (0, 3, 6, and 12 nmol P_i_) was determined in duplicates for each measurement. Note: The malachite green assay is a colorimetric method for measuring P_i_ in aqueous solutions and was performed on aliquots taken from the reaction tube. As such, it does not interfere with photoswitching. Conversely, the azobenzene has no absorption beyond 600 nm, which could potentially falsify the assay.

### Inhibition of ATP hydrolysis activity by resveratrol and PIAS 1–4

PIAS 1–4 stock solutions (concentrations: 0.01, 0.1, 0.5, 5, 10, 20, and 50 mm, dissolved in methanol) and resveratrol (concentration 0.01, 0.1, 0.5, 5, 10, 50, and 100 mm (Sigma‐Aldrich, D) in pure ethanol were diluted to 1% (v/v) and added to the *Y. lipolytica* ATP synthase sample (1 mg·mL^−1^ diluted 1/100 in reaction buffer) and incubated at room temperature for 1 h. As a control, 1% (v/v) of methanol/ethanol was added only. The ATP hydrolysis activities were then determined using the malachite green assay. To next study the effect of UV irradiation on the samples containing the azo‐compounds (absorption maxima at 365 nm) and their effect on the change in ATP hydrolysis activity due to UV irradiation, the UV irradiation experiments were performed the following way: a 365‐nm laser [Thorlabs (Dachau/Munich, Germany) M365L2‐UV (365 nm) mounted LED, 700 mA, 190 mW (min)] was used to constantly irradiate the reaction mix in each sample in the cuvette at a distance of 91.4 mm in a homemade setup as shown in Fig. S1. The irradiation time for the whole reaction time is 4 min. After that, the malachite green assay was performed the same as described for nonirradiated samples. Table S1 shows the results of each measurement before and after irradiation.

### Statistical analysis

The results (Fig. 3) were represented as the mean ± SEM of the three replicates from three independent experiments (*n *=* *9). A calibration curve for the absorption/P_i_ was generated to calculate the P_i_ concentration at each time point (P_i_/time) from the absorption of the malachite green complex at 620 nm (absorption/time). The P_i_ concentration per time corresponds to the ATP hydrolysis activity in Units/min. The percentage of inhibition was determined by normalizing the ATP hydrolysis activity of solutions with inhibitors with the native, monomeric *Y. lipolytica* ATP hydrolytic enzyme's activity. The values of the inhibitor concentration at which 50% of the ATP hydrolysis activity was inhibited (IC_50_ values) were calculated using graphpad prism® 5, version 5.01 (GraphPad Software, Inc., San Diego, CA, USA) by plotting the log concentration of the azo compounds versus the percentage inhibition of ATP hydrolysis activities.

## Results

### Design, synthesis and photophysical characterization of the PIAS

The design of the PIAS was based on an X‐ray crystal structure of the bovine mitochondrial ATP synthase in complex with resveratrol, piceatannol, and quercetin (Fig. 1). According to this structure, the polyphenols bind in a hydrophobic pocket between the rotor γ subunit C‐terminal end and the surrounding region formed by the stator α and β subunits (Fig. 1A, 36). This interaction blocks the rotation of the rotor against the stator and thereby catalysis. Accordingly, we reasoned that the PIAS inhibit the ATPase in their *trans* form, which closely resembles the polyphenols, and remain inactive in their bent *cis* form (Fig. 1B), which could not be accommodated in the sleeve‐like binding site defined by the X‐ray crystal structure.

The synthesis of PIAS 1–4 by azo coupling is shown in Fig. 1C. Diazotization of 3,5‐dimethoxyaniline (**1**) gave the diazonium salt **2**, which was treated *in situ* with the sodium salt of either phenol or guaiacol to give PIAS‐2 and PIAS‐3 in 75% and 70% yield, respectively. Global demethylation using excess boron tribromide in CH_2_Cl_2_ yielded PIAS‐1, an azolog of resveratrol, and PIAS‐4, an azolog of piceatannol, in 64% and 32% yield, respectively. PIAS 1–3 had previously been prepared by a similar sequence 29.

All four compounds (≥ 95% pure by ^1^H NMR) showed comparable absorption maxima between 340 and 360 nm in acetonitrile/water solution. No isomerization to the *cis* isomer was apparent upon irradiation with 365 nm even with high‐power LED light. This is due to the known very fast thermal relaxation of azobenzenes that bear a *para*‐hydroxy group 37, 38. In the dark as well as under ambient light conditions, we could only observe the *trans* isomer using ^1^H NMR spectroscopy. The photostationary states of the PIAS under physiological conditions could not be determined directly for the same reason.

### Choice of *Yarrowia lipolytica* ATP synthase as test system

The crystal structure of *trans*‐resveratrol and *trans*‐piceatannol in complex with the bovine F‐type ATPase shows a distinct binding pocket for polyphenols formed between an α‐subunit, the β_TP_‐subunit and the C‐terminal part of the γ subunit (Fig. 1A, 36). The residues of the binding pocket of polyphenols in the bovine enzyme are well conserved both within eukaryotic and bacterial types of ATP synthases (Fig. S2, alignment), suggesting that they harbor an identical binding pocket in the *Y. lipolytica* ATP synthase except for three amino acids in the γ subunit of which two (γI263V and γE264D) are conservative, and in the case of γK260N, the interaction occurs via hydrophobic interactions, while the positively charged nitrogen points away from the resveratrol molecule. Given that and the fact that the purification procedure of *Y. lipolytica* ATP synthase was already available in our laboratory 8, we decided to use this ATPase as a model system to study the effect of the PIAS on the ATPase hydrolytic activity. First, the fully assembled, monomeric form of *Y. lipolytica* ATP synthase was purified (Fig. 1D–F). Then, the ATP hydrolysis activity of the enzyme was determined to be 7 U·mg^−1^ with a variance of 3 U·mg^−1^ due to three biological replicates measured. The measured activities are well in the range of reported literature values 8. To test the F_1_–F_o_ coupled activity of the enzyme, we used the inhibitor oligomycin, which showed that 95 ± 5% of this ATPase's activity could be inhibited 39.

### Inhibition of *Y. lipolytica* ATP hydrolysis activity by resveratrol and *trans‐*PIAS 1–4

**Figure 2:**
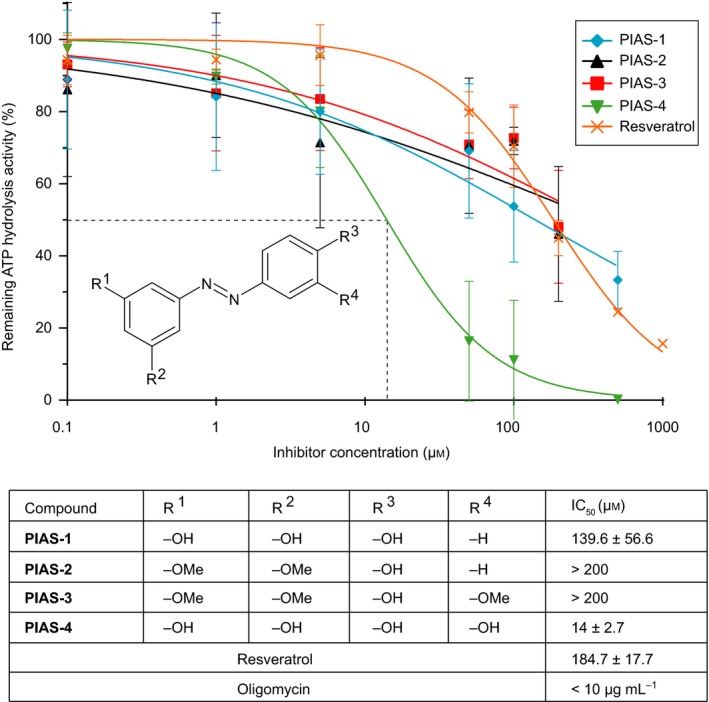
Inhibition of ATPase activity of *Yarrowia lipolytica* F_1_F_o_‐ATP synthase by PIAS 1–4. The inhibition of the ATP hydrolysis activity by PIAS 1–4 was determined using the malachite green assay. The results of the ATP hydrolysis activity measurements were plotted and fitted against the inhibitor concentration. The IC_50_ values are listed below in the table. Each measurement was done in triplicates and three biological replicates.

Using the fully active, coupled ATP synthase from *Y. lipolytica*, we next tested the capability of resveratrol and resveratrol derivates, PIAS 1–4, to inhibit the ATPase activity in a concentration‐dependent manner and with all compounds in their *trans* form (Fig. 2). First, the control experiment was performed, using resveratrol at concentrations from 0.1 to 1 mm. Resveratrol was able to reduce the ATP hydrolytic‐specific activity to 1.4 U·mg^−1^, corresponding to 16% of the initially uninhibited activity. The inhibition experiments were then performed using different concentrations from 0.1 to 200 μm (PIAS‐1, PIAS‐4) and 0.1 to 500 μm (PIAS‐2–3) and the inhibitory concentration that inhibits 50% of the initial activity (IC_50_) was calculated and determined, showing an IC_50_ of 184.7 ± 17.7 μm for resveratrol in the *Y. lipolytica* ATP synthase.

Furthermore, all four PIAS showed a concentration‐dependent inhibitory effect on ATP hydrolysis activity, however to various degrees. Among these derivatives, PIAS‐4, the azo‐polyphenol with an additional hydroxy group (R4=OH) was the most potent inhibitor, showing 100% ATPase inhibition at a concentration of 500 μm (IC_50_ = 14 ± 2.7 μm). PIAS‐1, the azo‐resveratrol derivative, was less potent and inhibited the ATPase with an IC_50_ = 139.6 ± 56.6 μm. The methylated derivatives PIAS‐2 and PIAS‐3, finally, showed the lowest inhibitory activity, with an IC_50_ above 200 μm in both cases.

### Optical control of ATP synthase activity *in vitro*

**Figure 3:**
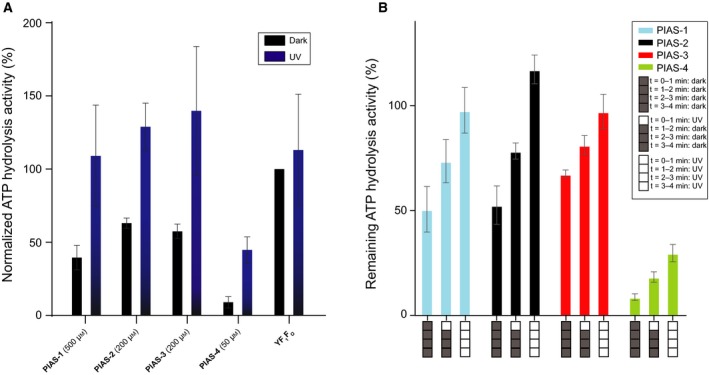
Optical control of ATPase activity *in vitro* using purified *Yarrowia lipolytica* ATP synthase (A) and reversibility (B). For each measurement, a sample of 0.01 mg·mL^−1^ ATP synthase in the reaction buffer was used to measure the initial ATP‐hydrolytic activity. The activities were normalized against the *Y. lipolytica* F_1_F_o_ ATPase activity under dark conditions. The ATPase activity was inhibited by adding (1) 500 μm, (2) 200 μm, (3) 200 μm, (4) 50 μm of compound PIAS‐1, 2, 3, and 4, respectively. All concentrations used were higher than the previously determined IC_50_ for each compound. The reactions were activated by switching UV laser light (365 nm) for 3 min at room temperature. The ATP hydrolysis activity was determined by the malachite green assay and normalized against the ATP hydrolysis activity of the native enzyme without UV irradiation. All compounds showed an inhibitory effect on ATP hydrolysis activity under dark conditions, which could be reduced by UV‐A irradiation. The assays were repeated four times and performed in triplicates. (B) For each inhibitor, measured series were performed under dark conditions (indicated by four black boxes), 1‐min UV irradiation followed by 3‐min dark conditions (one white box, three black boxes) and continuous UV‐A irradiation (four white boxes). The measurement was performed similar to the UV measurement shown in Fig. 3A, apart from the UV irradiation times. The ATP hydrolysis activity was determined by the malachite green assay and normalized against the ATP hydrolysis activity of the native enzyme without UV irradiation. All compounds showed inhibition of ATP hydrolysis activity under dark conditions, higher activities with 1‐min UV irradiation and the highest activity in case of 4‐min UV irradiation. The assays were repeated three times and performed in triplicates.

The PIAS derivatives undergo an isomerization from the stable *trans* isomer to the *cis* isomer upon irradiation with UV‐A light (Fig. 1B,C). Next, to study the impact of the *cis* isomers on ATPase, we used a 190 mW (Min) UV‐A laser at a wavelength of 365 nm to irradiate the samples for 3 min and determined their ATP hydrolysis activity shortly thereafter (Fig. S1). The PIAS concentrations were chosen (PIAS‐1: 500 μm, PIAS‐2–3: 200 μm, and PIAS‐4: 50 μm) according to their previously determined IC_50_ values. The chosen concentrations should ensure inhibition up to 60% while not blocking ATP hydrolysis completely, to be able to observe photoswitching effects (Fig. 3A). While the nonirradiated PIAS‐1–4 inhibited the *Y. lipolytica* ATP hydrolysis activities to different degrees, the samples irradiated with 365 nm light showed a reduced capability to inhibit the ATPase hydrolytic activity, comparable to the yeast wild‐type sample (compare purple columns of PIAS 1–4 with YF_1_F_o_ in Fig. 3A). Specifically, the nonirradiated PIAS‐4 inhibited ATP hydrolysis activity best and up to 90% at 50 μm after 3 min (black column of PIAS‐4 in Fig. 3A), while irradiated for 3 min, PIAS‐4 lost its potency for inhibition and showed only 55% of ATP hydrolysis inhibition (purple column of PIAS‐4 and YF_1_F_o_ in Fig. 3A). Remarkably, while all other three PIAS compounds (PIAS‐1, PIAS‐2, and PIAS‐3) were less potent in ATPase inhibition, they were able to regain the full level of wild‐type activity, showing no more inhibitory effect (purple columns of PIAS‐1–3 compared with YF_1_F_o_ in Fig. 3A). Hence, the 3‐min irradiation procedure completely abrogated their capability to inhibit ATPase.

### ATPase activity can be reversibly controlled by light

Next, we determined to what extent the described effect of PIAS 1–4 on ATP hydrolysis activity can be reversed to restore ATPase activity (Fig. 3B). To test this, we used an irradiation protocol that was applied on three differently treated 1 μg samples of *Y. lipolytica* ATPase: the first sample was kept in the dark for 4 min, the second sample was irradiated for just 1 min followed by 3 min in the dark, and the third sample was irradiated continuously for 4 min. After these treatments, the ATPase activity was determined (Fig. 3B). Generally, all compounds, PIAS 1–4, showed the lowest effects on ATP hydrolysis activity while being in the dark (Fig. 3B; 0–4 min: dark) but the highest ATPase activities upon continuous UV irradiation (Fig. 3B; 0–4 min: UV). Remarkably, ATP hydrolysis activity for the measured samples with 1‐min irradiation (Fig. 3B; 0–1 min UV, 1–4 min: dark) is lower than the activity for the measured samples with 4‐min irradiation time, which exemplifies the reversibility of ATPase inhibition using PIAS 1–4.

The observed results can be rationalized the following way: The PIAS 1–4 compounds in the *cis* conformation do not inhibit the ATP hydrolysis activity during the first minute of irradiation; however, they all isomerize back into their *trans*‐isoforms under dark conditions. It is this *trans*‐isoform in which PIAS 1–4 are again capable of inhibiting the enzyme. The differences in ATP hydrolysis activities for the three measured samples using PIAS 1–4 are due to their different potency to inhibit the ATPase: PIAS‐4 inhibits up to 90% under dark conditions, ~ 80% with 1‐min irradiation and 65% with continuous irradiation, while the nonirradiated PIAS 1–3 inhibit between 40% and 50%, between 20% and 30% with 1‐min irradiation and do not show any inhibition effect under continuous irradiation.

## Discussion

This study reports about the synthesis of new, reversible photoswitches derived from natural stilbenoid polyphenols such as resveratrol, for the optical control of the yeast *Y. lipolytica* ATPase activity. Resveratrol itself can be photoisomerized, but this requires short, cytotoxic wavelengths (< 300 nm) and eventually results in the formation of a photochemical byproduct (resveratrone) 40. We therefore synthesized four azobenzenes, PIAS 1–4, and evaluated them for their ability to reversibly inhibit ATPase activity, with resveratrol as reference inhibitor. The *trans*‐isomers indeed inhibit the ATP hydrolytic activity, while isomerization to the *cis* isomers decreased enzymatic activity inhibition. The study also demonstrates that PIAS‐1–4 can be used as reversible ON/OFF switches for *Y. lipolytica* ATPase.

**Figure 4:**
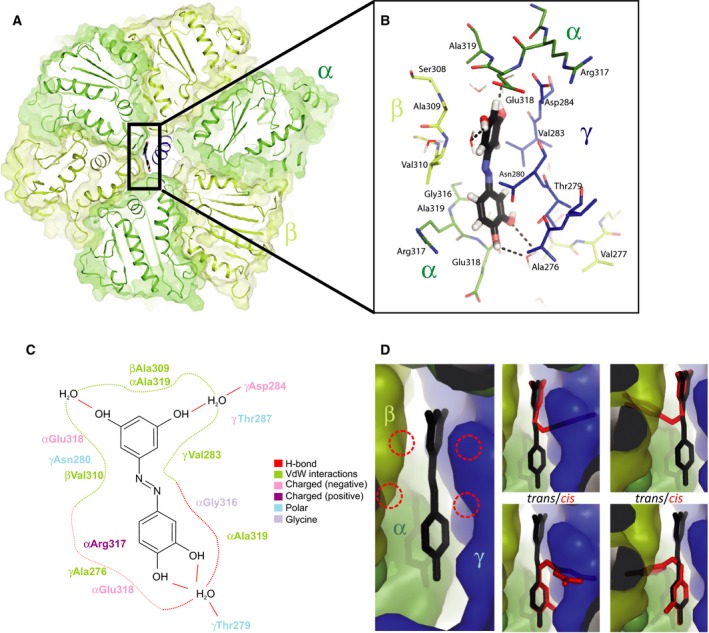
Model PIAS‐1 bound to the *Yarrowia lipolytica* F_1_ ATPase domain. The bovine F_1_‐resveratrol structure (pdb ID 2jiz, 36) was used to create a model with the *Y. lipolytica* ATPase (pdb ID 5fl7, 8) and the azo‐polyphenols used in this study. Colors: α dark green, β light green, γ blue) (A) Tilted view into the F_1_‐ATPase upward from the inner mitochondrial membrane. The azo‐resveratrol (stick representation, highlighted by a black box) binds in a pocket made by the subunits α, β, and γ. (B) Zoom [boxed in (A)] to interaction site of azo‐polyphenol bound to the F_1_‐ATPase. Hydrogen bonds are indicated by black dashed lines. (C) Two‐dimensional interaction plot of the azo‐polyphenol interaction with the F_1_‐ATPase (created using Maestro 41). Interaction distances are color coded. The interaction network of the polyphenol hydrogen bonds as well as hydrophobic and polar interactions contribute to the binding affinity of the polyphenol. (D) Modeling of *cis*‐ and *trans*‐polyphenols in F_1_ ATPase. Left panel: Side view of resveratrol‐F_1_ binding pocket. Various orientations of *cis*‐polyphenols (in red) are modeled and shown in the four right panels along with the *trans*‐configurations (black). All *cis*‐molecules generate steric clashes with the Van der Waals radii of the F_1_ subunits, indicated by the dashed circles in the left panel.

The high‐sequence homology between bovine and yeast *Y. lipolytica* ATPase and the isosteric nature of resveratrol and *trans*‐PIAS‐1 suggests that the molecular mechanism of binding and ATPase inhibition of the two compounds is identical. To support this notion, we further explored this possibility with molecular modeling using Maestro (41, Fig. 4C). The template yeast F_1_‐PIAS‐4 bound structure used for the molecular modeling was created in Pymol 42 by merging the bovine F_1_‐resveratrol structure (pdb ID 2jiz, 36) with the *Y. lipolytica* ATPase (pdb ID 5fl7, 8) and PIAS‐4. Our model of *trans*‐PIAS‐4 bound to the *Y. lipolytica* enzyme shows hydrophobic interactions of the azobenzene with residues from three different subunits of the F_1_ complex. The residues γV283, β_TP_V310, and β_TP_A309 are equivalent to residues γI263, β_TP_V279, and β_TP_A278 in bovine F_1_. Additionally, nonpolar interactions are formed by side chains that involve the two residues αA319 and γA276. The *trans*‐PIAS‐4 binding to the *Y. lipolytica* F_1_ complex appears to be further stabilized by a hydrogen bond network involving the hydroxy groups of *trans*‐PIAS‐4 and F_1_ complex intrinsic water molecules. It has been shown in purified *Escherichia coli* F_1_ and F_1_F_o_ ATPase in membrane vesicles that the relative positions of hydroxy groups of polyphenols appear to be critical for the degree of inhibition of ATPase hydrolysis 43. The four compounds PIAS 1–4, which have either methoxy or hydroxy groups at positions 1–4 inhibited the *Y. lipolytica* ATP synthase to different degrees. Given that and the findings made for the *E. coli* ATPase, we therefore suggest that the bulkier methoxy groups of PIAS‐2 and PIAS‐3 at positions 2 and 3 provide a rationale to understand the decreased inhibitory effects on *Y. lipolytica* ATP synthase by these two compounds. Their larger side chains may cause steric hindrance in the inhibitor‐binding side (Fig. 4D). Without further experimental structural information available, one cannot exclude the alternative possibility that decreased inhibition results from the absence of hydrogen bond donors at the ligand sites R1, R2, and R4.

While our studies were ongoing, Hoersch published the optical control of *E. coli* F_1_‐ATPase using a photoswitchable cross linker 44. Crosslinking with an azobenzene bismaleimide between engineered cysteines in the α‐ and β‐subunits reduced the ATP hydrolysis activity in a light‐dependent fashion. This approach requires genetic engineering of the ATPase and covalent attachment with maleimides. In contrast, our study uses the complete and genetically unmodified, native F_1_F_o_ ATP synthase holoenzyme (Fig. 1D–F); hence, it has the potential to be used with broader applicability, for example, in genetically nonmodified host cells.

Finally, our work extends the reach of photopharmacology to an important new target class and provides a blueprint for the development of photoswitches that enable to spatiotemporally control ATP‐dependent reactions, for example, in *in vitro* biotechnological applications. The exact mechanism by which resveratrol promotes a wide range of beneficial effects in humans is still unclear. As PIAS 1–4 inhibit ATPase in an analogous fashion, future photochemical experiments with the PIAS on other resveratrol targets and pathways such as AMPK and SIRT1, which are the key metabolic effectors of resveratrol, could provide more insights on the fundamental biochemical actions of resveratrol. This work could be explored to other resveratrol targets such as cyclooxygenases 45, phosphodiesterases 46, and estrogen receptors 47. PIAS‐1 and PIAS‐4 provide a proof of principle and a basis for further chemical modifications that potentially can fulfill the requirements of *in vivo* studies, for example, high‐binding affinities and mitochondrial‐targeting motifs. As the studied PIAS activities can be switched off, future work could shed more light on the molecular mechanisms governing the biochemical properties of resveratrol.

## Author contributions

TM and DT conceived and directed the study. BE designed and carried out biological experiments and analyzed data; FH designed and carried out chemical syntheses. All authors contributed to writing the manuscript.

## Supporting information


**Fig. S1**. UV irradiation experiment setup.
**Fig. S2**. Resveratrol binding site in the ATP synthase F_1_ complex.
**Fig. S3**. ^1^H NMR spectrum of PIAS‐4.
**Fig. S4**. ^13^C NMR spectrum of PIAS‐4.
**Table S1**. Reversible optical control of ATPase *in vitro* using purified *Y. lipolytica* ATP synthase.
