# Supplementary material for: Reversible optical control of F1Fo‐ATP synthase using photoswitchable inhibitors
Source: FEBS Lett. 2018 Feb 1;592(3):343–55. doi: 10.1002/1873-3468.12958 (PMC6175411; doi:10.1002/1873-3468.12958)
Supplement: Supplementary file 1 — Fig. S1. UV irradiation experiment setup. Fig. S2. Resveratrol binding site in the ATP synthase F1 complex. Fig. S3. 1H NMR spectrum of PIAS‐4. Fig. S4. 13C NMR spectrum of PIAS‐4. Table S1. Reversible optical control of ATPase in vitro using purified Y. lipolytica ATP synthase. [file FEB2-592-343-s001.pdf]

# Supplementary material for:

## Reversible Optical Control of $F_1F_o$-ATP Synthase Using Photoswitchable Inhibitors


Bianca Eisel[1,2,#], Felix Hartrampf[3,#], Thomas Meier[1,2,*], Dirk Trauner[3,4*]

[1]*Department of Structural Biology, Max Planck Institute of Biophysics, Max-von-Laue-Straße 3, 60438, Frankfurt am Main, Germany*

[2]*Department of Life Sciences, Imperial College London, Exhibition Road, London SW7 2AZ, United Kingdom*

[3]*Department of Chemistry, University of Munich, Butenandtstraße 5-13, D-81377 Munich, Germany*

[4]*Department of Chemistry, New York University, 100 Washington Square East, Room 712, New York, NY 10003*

## Supplementary figures

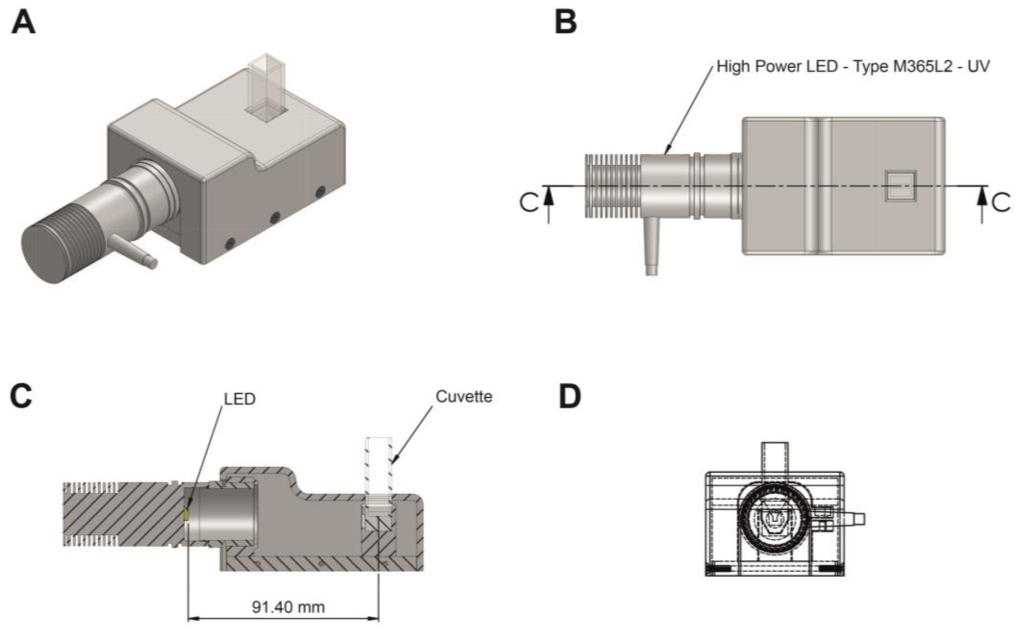


**Fig. S1.**

α

| | | |
|---|---|---|
| *B. taurus* | 285 | LRRPPGREAYPGDVF |
| *Y. lipolytica* | 311 | LRRPPGREAYPGDVF |
| *E. coli* | 276 | LRRPPGREAYPGDVF |

β

| | | |
|---|---|---|
| *B. taurus* | 272 | LGRIPSAVGYQPTLA |
| *Y. lipolytica* | 303 | LGRIPSAVGYQPTLA |
| *E. coli* | 258 | LGRMPSAVGYQPTLA |

γ

| | | |
|---|---|---|
| *B. taurus* | 252 | RTRQAVITKELIEIISGAAAL |
| *Y. lipolytica* | 272 | RTRQAVITNELVDIITGASSL |
| *E. coli* | 266 | KARQASITQELTEIVSGAAAV |

**Fig. S2**

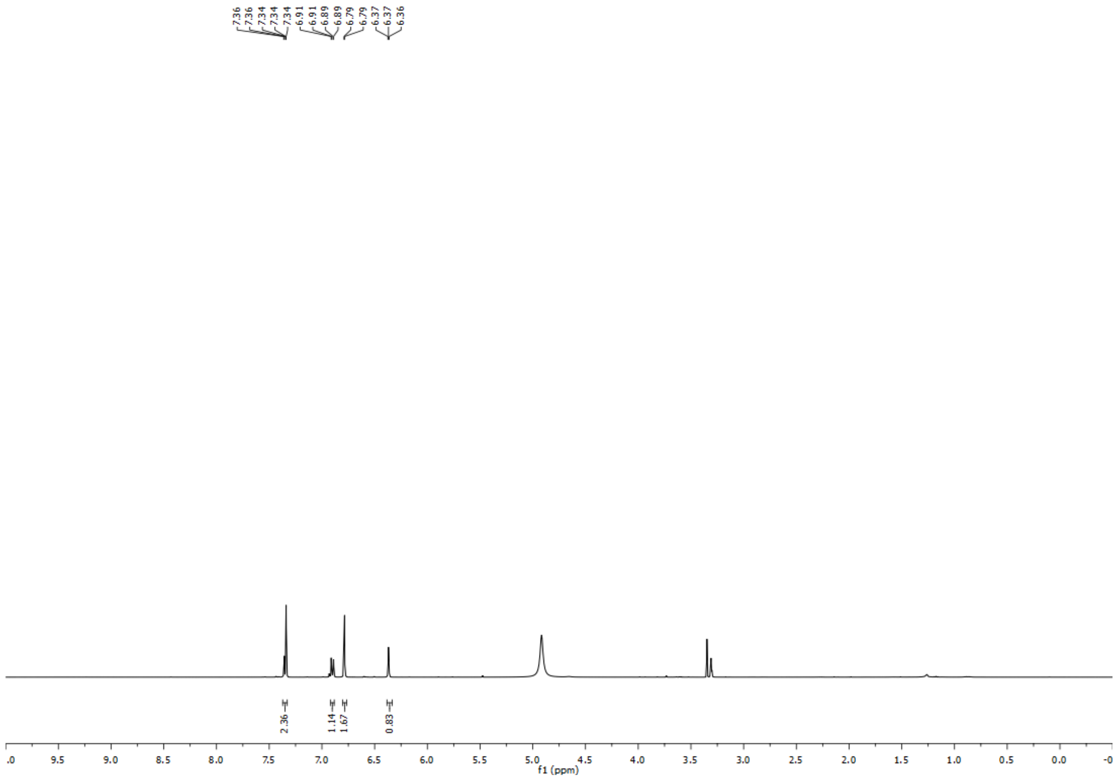


**Fig. S3**

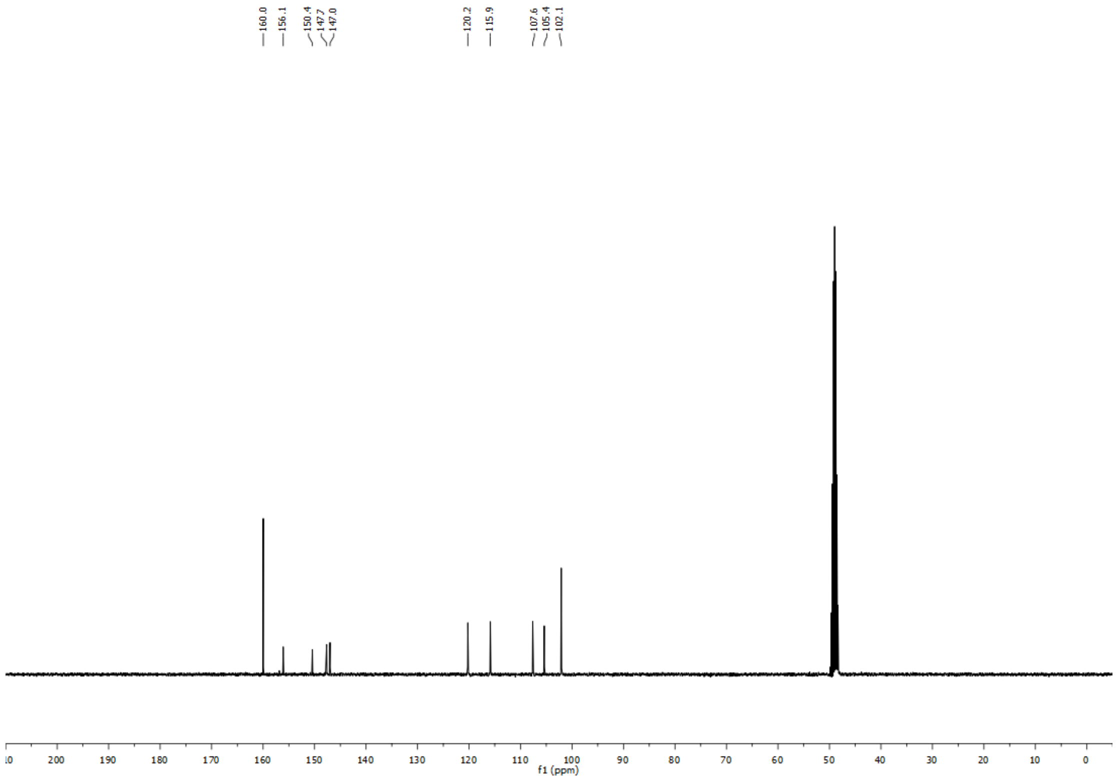
160.0
156.1
150.4
147.7
147.0
120.2
115.9
107.6
105.4
102.1
200
190
180
170
160
150
140
130
120
110
100
90
80
70
60
50
40
30
20
10
0
f1 (ppm)

**Fig. S4**

# Supplementary Tables

## Table S1.

| irradiation time [min] | ATP hydrolysis activities | | | | | | | |
|---|---|---|---|---|---|---|---|---|
| | **PIAS-1** | | **PIAS-2** | | **PIAS-3** | | **PIAS-4** | |
| | % | U/mg | % | U/mg | % | U/mg | % | U/mg |
| 0 | 50.6 ± 10.8 | 3.5 ± 0.4 | 56.8 ± 11.2 | 4 ± 0.4 | 65.1 ± 3.8 | 4.6 ± 0.2 | 8.8 ± 1.6 | 0.6 |
| 1 | 73.5 ± 10.3 | 5.1 ± 0.5 | 80.0 ± 4.5 | 5.6 ± 0.3 | 88.5 ± 17.3 | 6.2 ± 1.1 | 18.3 ± 2.5 | 1.3 |
| 4 | 97.7 ± 10.9 | 6.8 ± 0.7 | 114.3 ± 8.1 | 8 ± 0.6 | 101.1 ± 14.6 | 7.1 ± 1.0 | 29.8 ± 4.1 | 2.1 ± 0.1 |
